# Design and Validation of a Reflectarray Antenna with Optimized Beam for Ground Target Monitoring with a DVB-S-Based Passive Radar

**DOI:** 10.3390/s21165263

**Published:** 2021-08-04

**Authors:** Javier Rosado-Sanz, María-Pilar Jarabo-Amores, Jean-Yves Dauvignac, David Mata-Moya, Jérôme Lanteri, Claire Migliaccio

**Affiliations:** 1 Signal Theory and Communications Department, University of Alcalá, 28805 Alcalá de Henares, Spain; mpilar.jarabo@uah.es (M.-P.J.-A.); david.mata@uah.es (D.M.-M.); 2 Campus Sophia Tech. Laboratoire d’Electronique, Antennes et Télécommunications (LEAT), CNRS, Université Côte d’Azur, 06903 Sophia Antipolis, France; jean-yves.dauvignac@univ-cotedazur.fr (J.-Y.D.); jerome.lanteri@unice.fr (J.L.); claire.migliaccio@unice.fr (C.M.)

**Keywords:** antenna, reflectarray, passive radar, terrestrial targets, optimization, sectorial beam, simulation, measurements

## Abstract

A reflectarray antenna with an optimized sectorial beam is designed for the surveillance channel of a DVB-S-based passive radar (PR). The employment of satellite illuminators requires a high gain antenna to counteract the losses due to the great distance from the transmitter, but without forgetting a beamwidth wide enough to provide angular coverage. A method based on optimizing the position of several contiguous beams is proposed to achieve the required sectorial pattern. Different reflectarray elements are designed to achieve S-curves with smooth slopes and covering all the required phases (the S-curve represents the reflection phase of a single element, as a function of size, rotation and incidence angle). The real phase and modulus of the reflection coefficient of each element are considered in the optimization process to achieve the best real prototype. Geometry has been studied and adapted to employ commercial elements for the feed, feed-arm and the structure that holds the aperture. The designed prototype has been characterized in an anechoic chamber achieving a stable gain greater than 19 dBi in almost the complete DVB-S band, from 10.5 GHz to 12 GHz with a sectorial beam of $8.7^{\circ }\times 5.2^{\circ }$. The prototype has also been validated in PR trials in terrestrial scenarios allowing the detection of cars at distances up to 600 m away from the PR, improving the performance achieved with commercial parabolic dish antennas.

## 1. Introduction

A Passive Radar (PR) is a system that has the ability of detect and track targets without transmitting any electromagnetic signal, by taking advantage of the signals from other communication systems that are available in the scenario where the PR operates. The transmitter that emits that signal is called Illuminator of Opportunity (IoO). The IoO can be either from terrestrial communications systems or from satellite ones.

In PRs, the lack of devoted electromagnetic emission provides some useful advantages with respect to active radars, such as reduced cost and low power supply, which means portability potential, null electromagnetic pollution and low probability of interception. On the contrary, the exploitation of signals not designed for detection purposes and the null control of transmission parameters makes complex signal processing techniques necessary to detect the low energy scattered by the targets.

**Figure 1:**
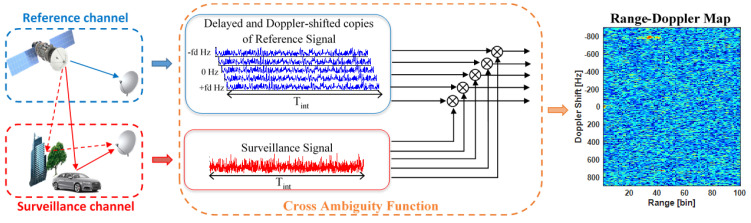
PR geometry scheme and operation principle.

The fact that a PR does not use a dedicated transmitter usually makes necessary the use of two acquisition channels: the reference one that acquires the direct signal from the IoO and the surveillance one that points towards the interest scenario and acquires the signal scattered by the targets (Figure 1). PR processing is based on the correlation of the surveillance signal with Doppler shifted copies of the reference one, which is called Cross-Ambiguity Function (CAF). The output of the CAF is a Range-Doppler (RD) map that usually defines the observation space for the detector design.

Satellite IoOs are being considered for PR operation in the last years due to their high availability and nearby global coverage [1]. DVB-S satellites are geostationary, which simplifies PR scenario geometry, their electromagnetic characterization and guarantees the availability of stable illuminating signals. DVB-S operates at frequencies in X and Ku bands (from 10.7 GHz to 12.75 GHz), which implies high propagation losses. Besides that, the transmitters are installed in satellites located at thousands of kilometres away from the Earth surface, so the power scattered from the targets is very low. In general, high gain surveillance antennas are required, especially if targets of interest are ground vehicles. The relation between the antenna gain and the radiation pattern defines a complex design problem: the generation of wide enough radiation beams for coverage sectors of interest in practical situations, providing the required gain to fulfil sensitivity requirements.

Characteristics of the signal and the PR geometry and desired performance define the main requirements of the antenna system: gain, beamwidth, frequency and bandwidth. Common solutions for DVB-S reception are based on commercial parabolic dish antennas, which provide high efficiency and gain but a narrow pencil beam [2,3,4]. Those antennas can fulfil coverage requirements for communications applications, but in radar the pencil beam radiation pattern limits the surveillance area. An alternative to commercial parabolic antennas, specially for these kind of applications, are reflectarray antennas.

A reflectarray is a planar surface composed of hundreds or thousands of printed elements (microstrip patches), each of which are pre-designed with a phase adjustment, in order to reflect the collimated electromagnetic field in a given direction, when it is illuminated by a feed [5]. These kind of antennas combine advantages from reflector antennas with the performance versatility of arrays, but avoiding the complex feeding networks [6]. Today, these antennas have become a really interesting solution due to their easy and low cost manufacturing employing the printed microstrip technology. They have been studied for different applications, mainly satellite communications and radar [7,8,9,10,11].

The design of a reflectarray comprises the following stages: feed design or selection and characterization, geometry design (feed position, focal length to diameter ratio f/D for maximizing efficiency, separation between array elements, and reflectarray shape), element selection and optimization, and phase distribution calculation [12].

The design of a sectorial beam reflectarray usually requires that the phase distribution is calculated through an optimization process, as there is not an analytical expression for it [13].

In [14], a method for designing a reflectarray antenna for the surveillance channel of a DVB-S-based PR was proposed. That method, employing an optimization for estimating the best positions of several contiguous beams, allows to achieve a sectorial beam for the detection and tracking of ground targets.

In this paper, that method is improved with the inclusion of a new variable in the optimization process that controls the influence of the phases driving the central broadside beam over the phases that generate the rest of contiguous beams. The improved method is employed to design an elliptical reflectarray that could employ the feed, feedarm and aperture holding structure from a commercial parabolic dish antenna, allowing a low cost and fast assembling. The geometrical parameters of the commercial set have been estimated and employed for the reflectarray design, driving to a 22×16 elements aperture. The feed radiation pattern was characterized in an anechoic chamber and modelled using the Ansys HFSS electromagnetic simulator for being included in the reflectarray antenna design and optimization.

The antenna was manufactured and validated through measurements in an anechoic chamber and with radar trials in two real scenarios. Results of anechoic chamber measurements show that the manufactured reflectarray generates a sectorial beam of $8.7^{\circ }$ in azimuth and $5.2^{\circ }$ in elevation with a quite stable measured realized gain greater than 19 dBi in the frequency range [10.5–12] GHz and a maximum of 19.82 dBi at 11.25 GHz. Results of radar trials show that the reflectarray antenna allows to detect and track small ground targets at distances up to 600 m from the PR, providing a great improvement in the PR demonstrator coverage with respect to previous versions that employed commercial parabolic dish antennas.

## 2. Design Method

The proposed method is based on the generation of a sectorial beam reflectarray from the combination of several contiguous ones. An optimization algorithm is employed to find the best steering of the beams, whose combination generates the sectorial pattern with the desired beamwidth. SideLobe Level (SLL) reduction is also considered for optimization. A particle swarm optimization (PSO) algorithm [15] is chosen for this purpose.

**Figure 2:**
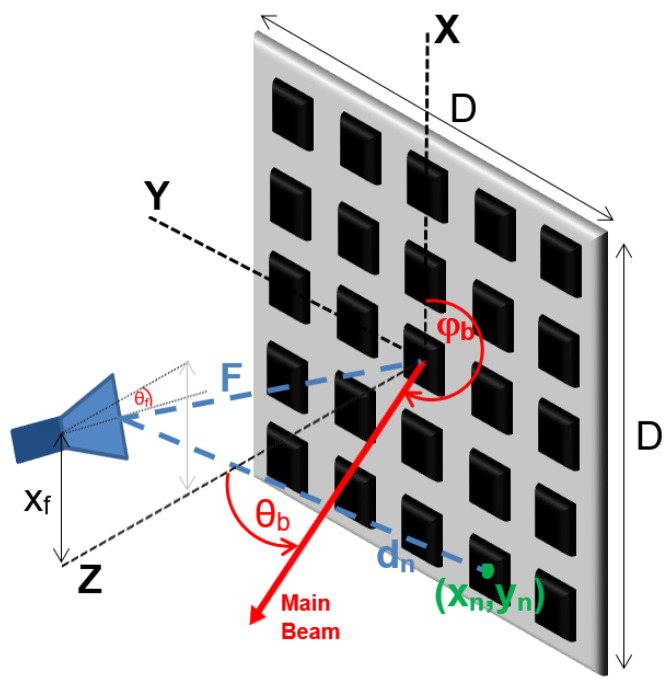
Main geometrical parameters of a reflectarray antenna.

Figure 2 defines the coordinate system and the geometry parameters employed in the design method. The radiation pattern (RP) of a reflectarray can be estimated through array summation from the feed pattern, the geometry knowledge and an accurate characterization of the reflection coefficients of the elements (Equation (1)). The calculus of the RP is more useful in UV coordinates, because the optimization techniques employed to achieve the required radiation distribution are based on the comparison of the pattern with some masks, which are easily defined in UV space. $E_{inc_{m,n}}$ is the incidence field from the feed at each element, $|s_{11_{m,n}}|$ the modulus of reflection coefficient of each element and $\phi_{m,n}$ the phase of the reflection coefficient at each element.

(1) $$E_{r}\left(u,v\right)=\sum_{m=1}^{M}\sum_{n=1}^{N}E_{inc_{m,n}}\left|s_{11_{m,n}}\right|e^{j\phi_{m,n}}e^{jk_{0}\left(ux_{m,n}+vy_{m,n}\right)}$$

The optimization vector or *particle* is composed of 4 variables. For the *k*-th iteration: $P_{k}=\left[v_{b1,k};v_{b2,k};A_{b0,k};\phi_{cent,k}\right]$. The first two variables of each particle are the *v* positions of the desired maximum radiation of two contiguous beams denoted as beams 1 and 2 (b1 and b2). The *u* position for all the beams is fixed by the desired steering of the final sectorial beam $\theta_{sa}$ (Equation (2)). It is expected that these two beams, together with the zero steering beam ($v_{b0}$) and the other two symmetrical beams at the zero left-side ($v_{b-1}$ and $v_{b-2}$), achieve a sectorial beam of the required beamwidth.

(2) $$u_{b}=sin\left(\theta_{sa}\right)$$

The required phases at each element $\phi_{R}\left(x_{n},y_{n}\right)$ to generate the sectorial beam are calculated as the weighted arithmetic mean of the phases required to generate each of the beams steering to $\left(\theta_{bi},\varphi_{bi}\right)$ (Equation (3)), being $tan\varphi_{bi}=\frac{u_{b}}{v_{bi}}$ and $sin\theta_{bi}=\sqrt{u_{b}^{2}+v_{bi}^{2}}$.

(3) $$\phi_{R}\left(x_{n},y_{n}\right)=\frac{\sum_{i=-2}^{2}A_{bi}\cdot k_{0}\left(d_{n}-\left(x_{n}cos\varphi_{bi}+y_{n}sin\varphi_{bi}\right)sin\theta_{bi}\right)}{\sum_{i=-2}^{2}A_{bi}}$$

The phase distribution $\phi_{R}\left(x_{n},y_{n}\right)$ is taken for calculating the radiation pattern of a reflectarray of *M* row elements by *N* column elements through array theory formulation [16]. The weights employed are: $A_{b0}$, obtained from the optimization process, for the phases that generate the zero steering beam, and those for the other 4 beam cases. $k_{0}$ is the wavenumber at operation frequency, $d_{n}$ the distance from feed to *n*-th element and $\left(x_{n},y_{n}\right)$ the position of the *n*-th element. The real phases achievable with the elements (ϕ), previously obtained by electromagnetic simulations, are employed, instead of ideal ones, to calculate the most reliable radiation pattern, as well as the losses ($|s_{11}|$) introduced by each element (Equation (1)). As it was probed in [14], the S-curve of the elements varies significantly from normal incidence $\Theta_{inc\_norm}=0^{\circ }$ to maximum incidence in the border elements, so the real phases and losses for a set of incidence angles comprising all the possible range are employed $\Theta_{\mathrm{inc}}=\left[0^{\circ },5^{\circ },10^{\circ },15^{\circ },20^{\circ },25^{\circ },30^{\circ },40^{\circ },50^{\circ }\right]$, assigning to each element the nearest angle of $\Theta_{\mathrm{inc}}$.

The fourth optimization variable $\phi_{cent,k}$ fixes the phase of the central element, shifting the phases of all the element in order not to change the phase distribution. Optimizing this variable allows having the different elements in the most efficient distribution according to their real losses.

In [14], the main beam-width and SLL were optimized only in two planes (azimuth and elevation), by employing two masks. In the present work, the cost function to be minimized has been redefined as expressed Equation (4), being the SLL value minimized in all the space (u,v).

**Figure 3:**
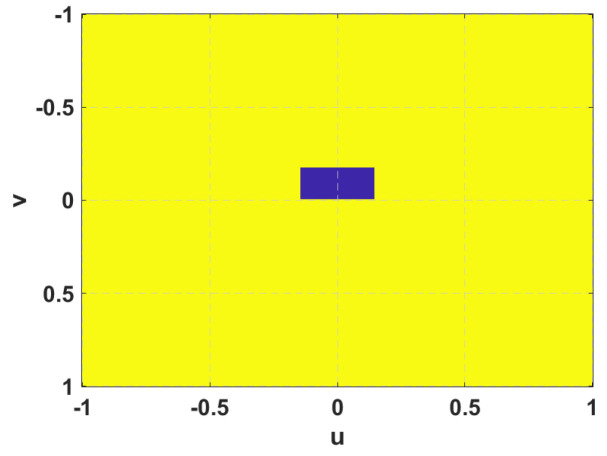
2D mask in UV space to calculate the SLL outside (yellow part) the main beam (blue part) area.

A mask in the azimuth plane ($mask_{inf}$) is employed to force the design to fulfil the required −3 dB beamwidth, but the SLL is estimated in all the space by means of a two-dimensional mask ($mask_{2D}$) that delimits the area outside the main beam, being defined by the beamwidths at −10 dB (Figure 3). Only the points of that area in which the radiation is above a determined level are computed in the cost function, trying to minimize the radiation outside the main beam. A pseudo-directivity ($\hat{dir}$) is considered as the ratio between the maximum radiation of the main beam and the mean radiation in the area outside the main beam (delimited by $mask_{2D}$). Three constants are employed to control the importance of each of the parameters in the optimization cost function: K${}_{1}$ for pseudo-directivity, K${}_{2}$ for azimuth beamwidth and K${}_{3}$ for SLL.

(4) $$C=-K_{1}\hat{dir}+K_{2}\sum RP_{azi}<mask_{inf}+K_{3}\sum RP_{2D}>mask_{2D}$$

where:

$$\hat{dir}=\frac{max(E_{r}\left(u,v\right))}{\bar{E_{r}}\left(u_{m},v_{m}\right)};\;\left(u_{m},v_{m}\right)\in mask_{2D}$$

$$RP_{azi}=\frac{E_{r}\left(u_{b},v\right)}{max(E_{r}\left(u_{b},v\right))};\;v\in \left[-1,1\right]$$

$$RP_{2D}=\frac{E_{r}\left(u,v\right)}{max(E_{r}\left(u,v\right))};\;\left(u,v\right)\in \left[-1,1\right]$$

## 3. Design Characteristics

### 3.1. Feed Characterization

**Figure 4:**
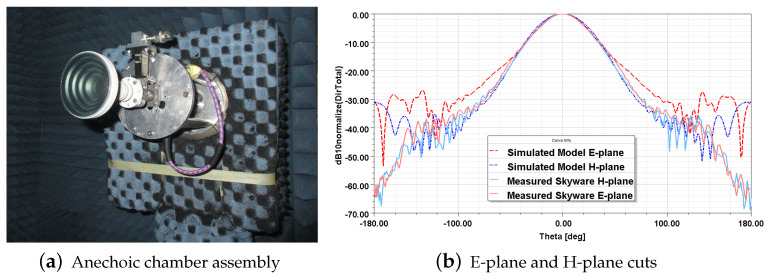
*Skyware* antenna assembly for characterization in anechoic chamber and its measured radiation pattern and the simulated one with the model created from measurements results.

The reflectarray will be fed with a commercial *Skyware* horn antenna. It is an axial corrugated horn in which the geometry’s parameters cannot be measured, as its aperture is covered by a protective plastic sheet, so it is difficult to model it in the electromagnetic simulator. In order to achieve an optimum design, the real aperture illumination must be taken into account. Therefore, the feed RP was obtained by measurements in the anechoic chamber of the *High Technology and Homologation Centre (CATECHOM)* of the University of Alcalá (Figure 4a), and it defines $E_{inc_{m,n}}$ in Equation (1) for the optimization process.

For the second stage, the check of performance through electromagnetic simulation and fine adjustments, the feed has been modelled as a *Potter* horn [17], because it provides an excellent radiation pattern with suppressed sidelobes, similar to the characteristics obtained in the measurement of the *Skyware* antenna. The Potter antenna is composed of different cylinder and conical sections that serve as a step transition in diameter. An optimization carried out for different diameters of these sections allowed us to find a horn model that minimizes the differences with the measured RPs, especially in the region of interest ($D_{norm}\geq -10$ dB). The comparison between the measured RP cuts and the simulated ones is shown in Figure 4b.

### 3.2. Geometry Selection

The commercial feed arm and aperture holding structures define the main geometrical parameters in the designed reflectarray antenna. In the geometry defined in Figure 2:

- The feed is located at position $X_{f}=138.7$ mm, $Y_{f}=7.1$ mm (due to feedarm misalignment) and $Z_{f}=179.7$ mm. This position implies a focal length F=227 mm. It has an angle of rotation $\theta_{f}=28.78^{\circ }$ with respect to the normal of the aperture.
- The feedarm limits physically the maximum semi-distance from the centre to one edge of the aperture in X axis dimension to $S_{x}=220$ mm.
- The focal length to reflectarray diameter ratio (F/D) is selected to maximize the aperture efficiency $\eta_{a}$ in Y axis dimension, while the X axis size ($D_{x}$) is increased from the one achieving maximum aperture efficiency in order to decrease the elevation beamwidth. According to the horn radiation pattern and focal length, the aperture efficiency in the normal plane to Y axis is maximum for $f/D_{y}=0.83$ ($\eta_{a}=68.6\%$), which means $D_{y}=273$ mm. $D_{x}$ is fixed at about 374 mm, achieving an aperture efficiency of $\eta_{a}=61.4\%$.
- The inter-element spacing is fixed to be 17 mm, so a reflectarray of 22×16 elements can be designed.

### 3.3. Element Selection

The element selection is a key step in reflectarray design, because it determines the reflection efficiency and the bandwidth of the antenna. The shape of each element must be selected and the change in phase must be characterized as a function of a change in dimensions or rotation, which is called the “S” curve of the element. This curve must cover a phase-shift of at least $\pm 180^{\circ }$ with a smooth slope [12].

**Figure 5:**
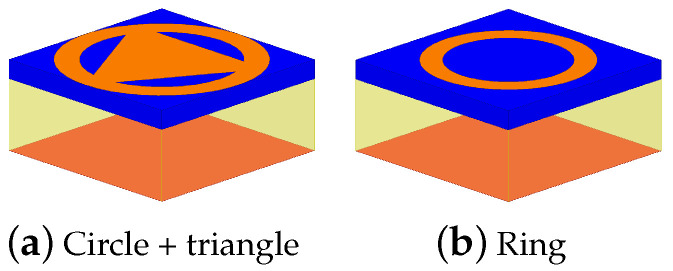
Elements selected for the design.

In order to cover all the needed range of phases, with a smooth slope, three different elements are employed: circle plus triangle and ring with two different widths $w_{ring}$ (Figure 5 and Table 1). The patch elements are located over an FR4 substrate 1.6 mm thick which is over a 6 mm thick air substrate. The ground plane is located below the air substrate.

**Figure 6:**
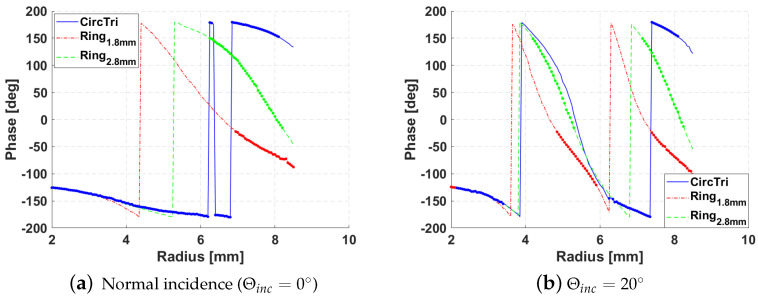
S-curve of selected elements for normal incidence and $\Theta_{inc}=20^{\circ }$, highlighting the valid ones in each case according to the maximum of the allowed losses.

The S-curves of the selected elements were simulated by unit cell analysis configured for horizontal polarization in the final design (Figure 6). The reflection phase varies significantly with the angle of incidence, so it must be taken into account in the reflectarray design process. On the other hand, there is a gap of non-covered phases within the range [−120,−90] (Figure 6a). The proposed designed method will solve this problem by not selecting these phases for the central elements.

## 4. Design Results

The proposed method is configured with the following requirements and parameters for cost calculation:

- Design frequency f=11 GHz and $\theta_{sa}=-5^{\circ }$;
- Azimuth 3 dB beamwidth: $10^{\circ }$. Elevation 3 dB beamwidth: $6^{\circ }$;
- Azimuth 10 dB beamwidth: $16^{\circ }$. Elevation 10 dB beamwidth: $9^{\circ }$;
- SLL: −20 dB;
- $K_{1}=0.1$; $K_{2}=2$; $K_{1}=10$.

**Figure 7:**
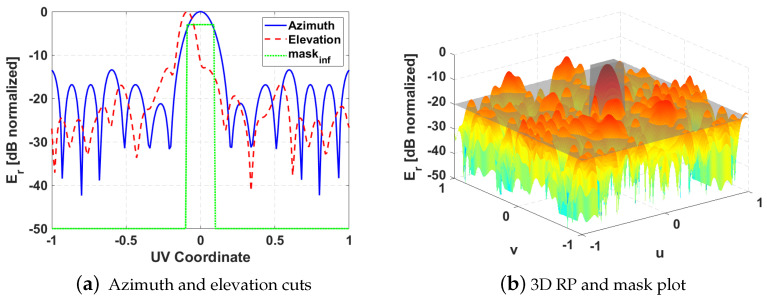
Results of the proposed optimization method launched in Matlab for reflectarray design at 11 GHz.

The method described in Section 2 gives the best cost result (C=9.1) for beam positions $\varphi_{b1}=0.0509$ and $\varphi_{b2}=0.0671$, zero-beam pondering as half ($A_{b0}=0.5$) and central element phase $\phi_{cent}=48.3^{\circ }$ (ring element of $w_{ring}=2.8$ mm). Azimuth and elevation cuts of the resulting RP are shown together with $mask_{inf}$ in Figure 7a. Figure 7b shows the 3D RP of the achieved design and the $mask_{2D}$ employed for SLL minimization.

**Figure 8:**
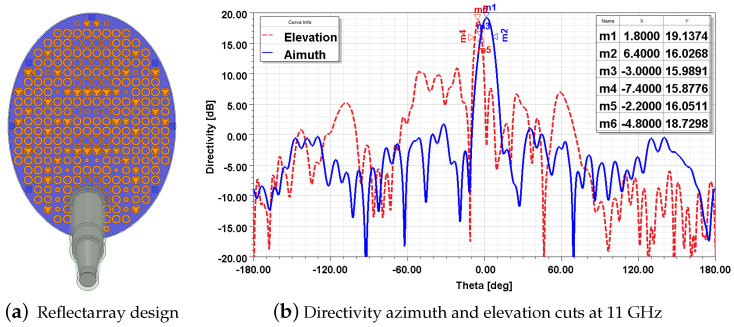
Reflectarray modelled in HFSS and simulation results showing RP cuts.

The phase distribution obtained in the optimization process was employed to create a reflectarray in ANSYS HFSS with elements described in Section 3.3. It has been simulated by employing the Finite Element-Boundary Integral (FE-BI) method, because the design is electrically large, and being fed with the *Potter* horn previously optimized to approximate the real one (Figure 8a). *Foam* spacers that will allow to have a thick air substrate between the *FR4* and the ground plane are also included in the simulation.

Simulation results are shown in Figure 8b. A 3 dB azimuth beamwidth of $9.4^{\circ }$ is achieved as well as $5.2^{\circ }$ elevation beamwidth, with a maximum directivity of 19.13 dBi achieved at $\left(\varphi ,\theta \right)=\left(1.8^{\circ },-4.8^{\circ }\right)$ from the broadside. High radiation levels appear in the elevation cut at angles between $-50^{\circ }$ and $-20^{\circ }$ due to the specular radiation from the feed. In [14], it was proposed to take advantage of this specular radiation by steering the main beam towards it, but in this case it is not useful for the radar application to have the main beam steered to $37^{\circ }$.

## 5. Prototype Characterization

The element distribution of the reflectarray aperture was transferred to an FR4 PCB through chemical etching. The reflectarray antenna structure was manufactured assembling the ground plane and the FR4 board that contains the patches, with foam spacers to generate the intermediate air substrate, and it was installed in the commercial holding structure and feedarm for horn location.

**Figure 9:**
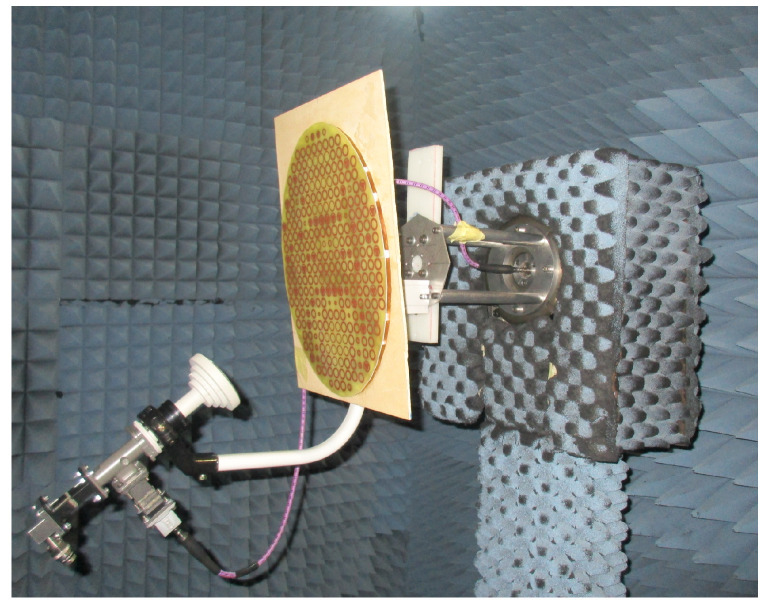
Manufactured reflectarray prototype assembly for characterization in the anechoic chamber.

Measurements were performed in the anechoic chamber of the *High Technology and Homologation Centre (CATECHOM)* of the University of Alcalá (Figure 9). Due to the dimensions of the anechoic chamber (3.6 m), the maximum diameter of the antenna (40 cm) and the measurement frequency (11 GHz), measurements were carried out in near field and transformed to the far field. The gain was estimated employing a standard gain antenna and applying the Friis transmission formula and the corresponding near-field to far-field conversion factors [18].

First, a near field elevation cut was made at design frequency (11 GHz) in order to characterize the antenna holding structure and to steer the main beam along the horizontal plane. After this calibration stage, full 3D RP measurements were carried out, for frequencies ranging from 10 GHz to 12.5 GHz.

**Figure 10:**
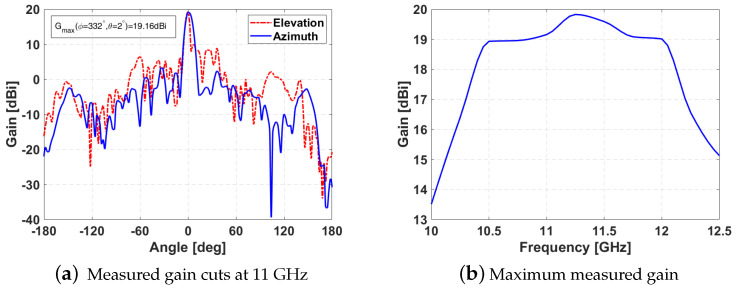
Reflectarray characterization results.

Measurements results are shown in Figure 10. The main cuts at the design frequency (Figure 10a) are similar to the simulated ones with beamwidths of $8.7^{\circ }$ in azimuth plane and $5.2^{\circ }$ in elevation. The maximum gain is above 19 dBi in the frequency range [10.5–12] GHz (Figure 10b), which means that the design antenna operates correctly in almost the full DVB-S band.

## 6. Validation in Real PR Trials

### 6.1. IDEPAR Demonstrator and Trials Scenario

IDEPAR is the PR technological demonstrator developed at the University of Alcalá [19]. The designed reflectarray is integrated in its surveillance channel for DVB-S PR trials while a commercial dish antenna of 80 cm diameter is employed in the reference channel to acquire the direct signal from the *Hispasat 30W-5* satellite. The horn of both channels is the same, as well as the LNBs to downsample the Ku signal to frequencies about 1.5 GHz that can be acquired by an *USRP X-310* board. The selected central frequency is 11.3 GHz with an acquisition bandwidth of 100 MHz covering 3 DVB-S channels: one of 30 MHz and two of 10 MHz.

**Figure 11:**
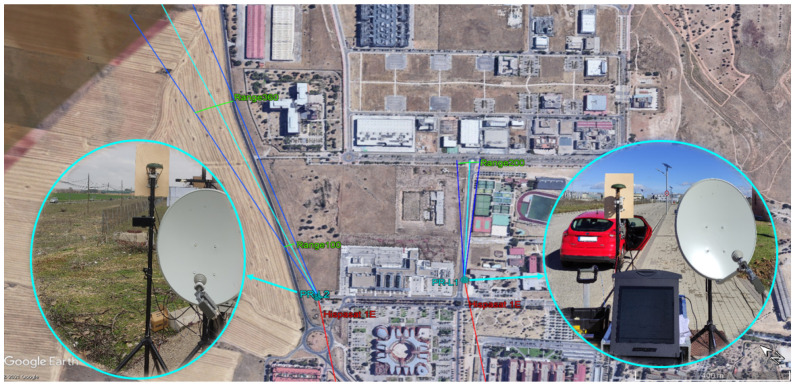
Terrestrial scenarios employed to validate the reflectarray antenna and the PR deployment in each of them.

To validate the reflectarray design, the IDEPAR demonstrator was deployed in two semi-urban terrestrial scenarios located in the External Campus of the University of Alcalá (Figure 11).

The first deployment is next to a Nursing School with its surveillance area covering a straight street surrounded by medium-height buildings, some trees and a metallic fence that surrounds the sports facilities. The main beam covers all the street up to its ends at range bin 200 (~330 m from the PR).

The second deployments is at the border of the campus, next to the *M*-121 road which will be the region of interest for ground traffic monitoring. In this scenario, there are some buildings on the right side of the road and crops on the left side. The beamwidth covers the road from range bins 100 to 360, in which a target in the road will be at 160 m and 600 m from the PR, respectively.

### 6.2. Target BRCS and Coverage Estimation

**Figure 12:**
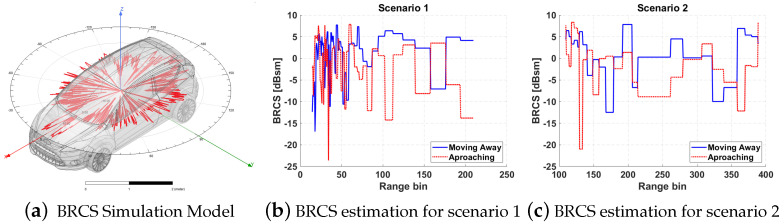
RCS simulation model and estimated BRCS in each range bin for the two trial scenarios in the two possible trajectories along the roads: moving away from the PR or approaching it.

The Bistatic Radar Cross Section (BRCS) of a model similar to the car employed as controlled target in the first deployment was simulated in ANSYS HFSS employing a multi-bounce rays approach with the physical theory of diffraction (PTD) correction. The RCS for all possible incidence and reflection azimuth angles $\phi \in \left[0,360\right]$ were simulated, fixing the elevation of incidence wave at $\theta_{inc}=55^{\circ }$ (Hispasat 30W-5 elevation) and the reflected one $\theta_{inc}=90^{\circ }$ because the targets and the PR are approximately at the same altitudes. Figure 12a shows the model employed for simulating the BRCS and, as an example, the results achieved along all reflected angles for incidence $\phi_{inc}=180^{\circ }$, that is incidence in the centre of the rear part of the car.

The possible trajectories along the two main roads of both scenarios were analysed to calculate the change in azimuth incidence and reflection angles along them. Knowing those angles, the car BRCS along the roads can be estimated (Figure 12b,c), showing that, for these geometries, this kind of targets reflect more from their rear part, when they are moving away from the PR, than from their front part, when they are approaching.

**Figure 13:**
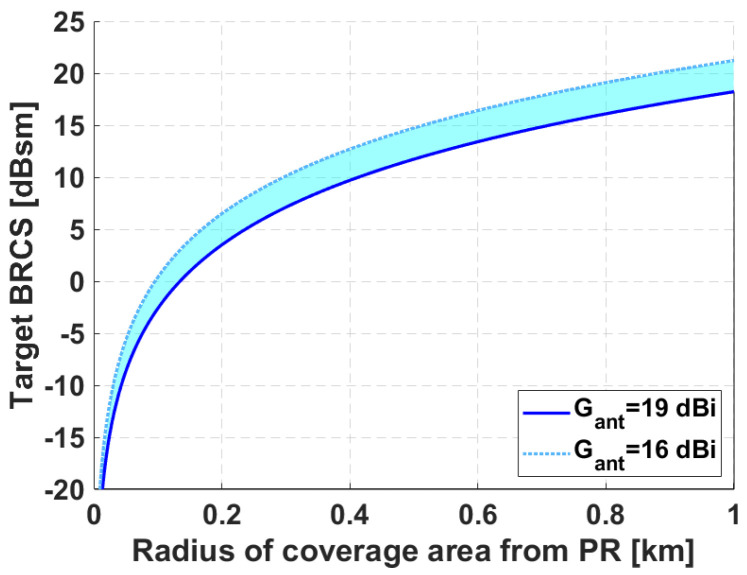
Curves of coverage ranges versus target BRCS for the measured antenna gain. System sensitivity equals−148.5 dBm calculated for $P_{D}=80\%$ and $P_{FA}=10^{-4}$ ($SNR_{DET}=16$ dB).

The coverage curves from the PR as a function of the target BRCS are shown in Figure 13. For coverage calculation, the sensitivity of the system was calculated from the theoretical Signal-to-Noise Ratio (SNR) required for detecting Swerling I targets with additive white Gaussian noise, and the noise factor of the receiver; propagation losses were also considered. The estimated angular coverage, in which the receiving antenna gain is in the range between the maximum measured and the one at the beamwidth edges ($G_{max}$-3 dB) is marked in Figure 11.

### 6.3. PR Trials Results

**Figure 14:**
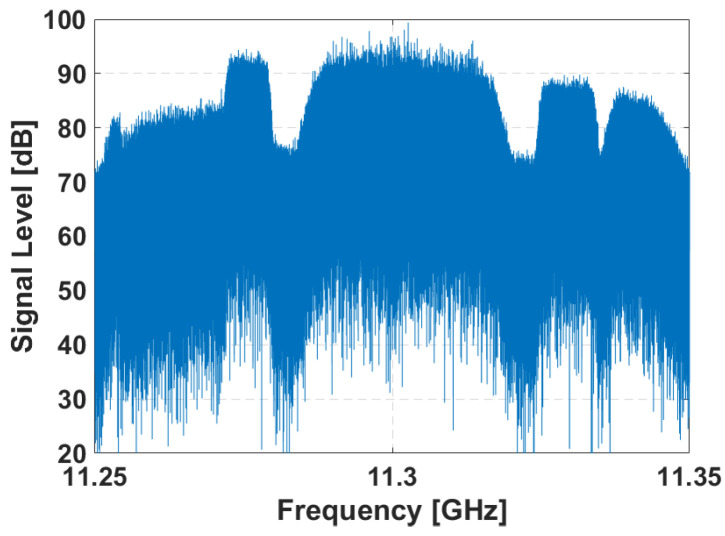
Spectrum of the signal acquired by the reference channel in one of the measurements of the trials.

Acquisitions in both validation scenarios were carried out at a central frequency of 11.3 GHz, covering three DVB-S channels horizontally polarized, as shown in Figure 14. The signals were filtered digitally, to eliminate the noise at frequencies where there is no DVB-S channel and an Extensive Cancellation Algorithm (ECA) [20] was employed to mitigate the Direct Path Interference (DPI), improving the target detection. The coherent processing interval (CPI) was fixed to 200 ms which produced a set of 75 RD maps from each measurement of 15 s.

**Figure 15:**
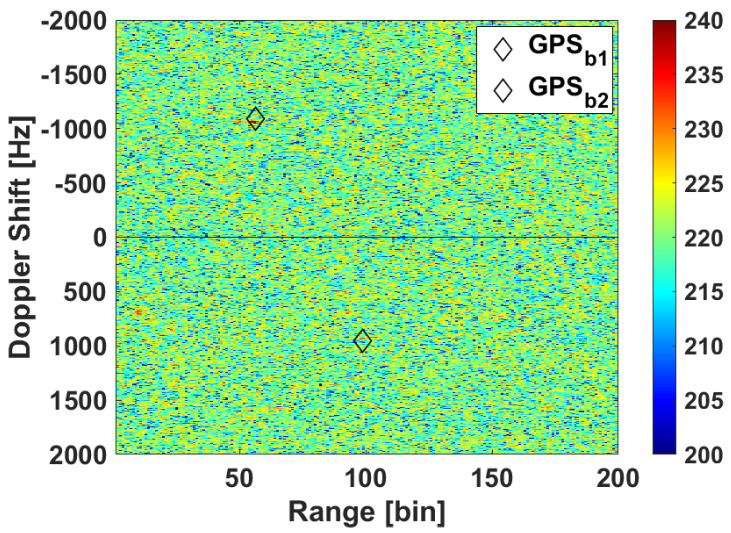
RD map of CPI 59 for validation of the designed reflectarray in scenario 1.

**Figure 16:**
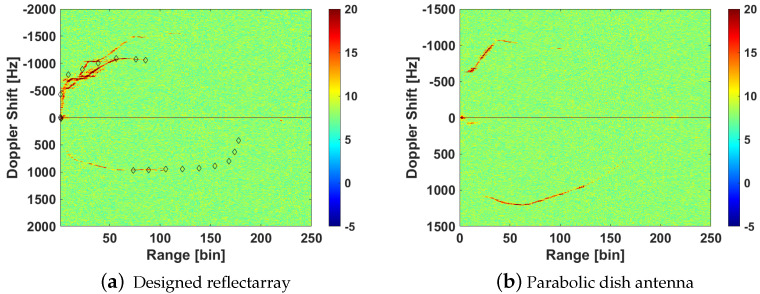
Cumulative RD maxima from results of trials in scenario 1 employing the designed reflectarray and commercial parabolic dish antenna in the surveillance channel.

The deployment in scenario 1 for the first trials with the reflectarray antenna was performed on the 10th of February of 2021. From all the measurements carried out, one is selected in which there were two cooperative targets: one of them moved away from the PR and the other started at the end of the road and moved towards the PR. Besides those, there were two more cars in the street at that moment, one in each direction. Figure 15 shows the RD map together with the GPS ground truth of cooperative targets for CPI 59. Figure 16a shows the cumulative of the maxima from all the 75 RD maps, each of them normalized by its pedestal noise level, and the GPS ground truth during all the acquisition. The conversion of GPS data to Range-Doppler is not precise when the target is accelerating, because a small error in sample time gives a great difference in the Doppler shift ($f_{D}$) due to the high operation frequency. Some targets, probably bigger than cars, can be detected crossing the round at the end of the street, at range bin around 220.

Figure 16a can be compared with the results published in [4] in which a commercial dish antenna of 40 cm diameter was employed for the surveillance channel in the same scenario (Figure 16b). Comparing the results of a car moving away in both cases, a great improvement with the reflectarray antenna can be observed, as the signal to interference ratio for the same kind of targets in range bins between 50 and 100 has increased considerably. Table 2 shows the quantitative comparison, in terms of the probability of detection $P_{D}$ and maximum detected range bin, for both acquisition in same scenario with reflectarray and parabolic dish as surveillance antennas, employing the same CA-CFAR detector configured for a desired probability of false alarm $P_{fa}=10^{-4}$, and a tracker stage based on the Kalman filter. $P_{D}$ is estimated as the ratio between true detections over the tracker-estimated positions. Analysing the case of a car moving away, the maximum detection range in the case of reflectarray is limited by the end of acquisition time, i.e., last CPI of acquisition contains a detection. In the case of the parabolic dish antenna, the maximum range bin where a detection is associated with the track is 144, but from range bin 58, the density of detections associated with the track is low, which explains the decrease in $P_{D}$.

**Figure 17:**
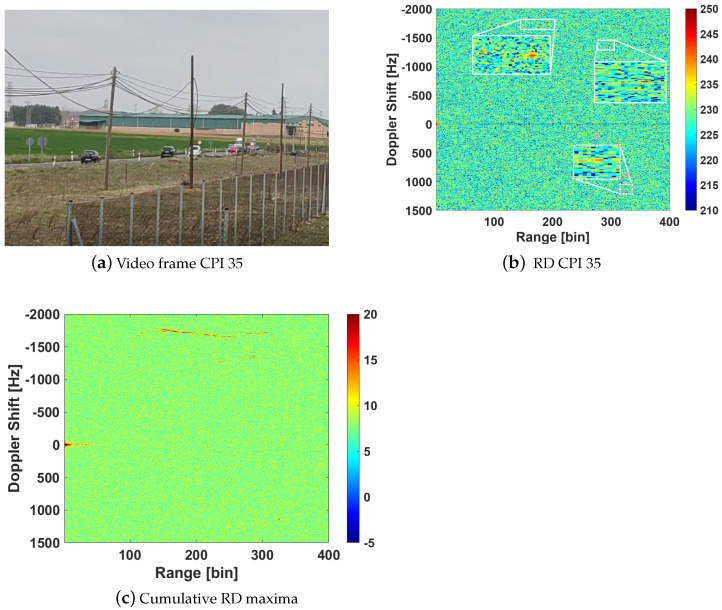
Video ground truth of an instant of the measurement in scenario 2, its corresponding RD map and the cumulative of RD maxima.

The deployment in scenario 2 was performed on the 2nd of March of 2021. In this scenario, there is usually a lot of traffic, so a controlled target was not employed. The following targets were checked by video ground truth (Figure 17a):

- **Moving away** from nearer to further from the PR: a car probably out of the main beam, two vehicles at range bins about ~150 and ~200 and $f_{D}$ ~ −1700 Hz (Figure 17b) and another set of 2 cars, one of which appears at range bin ~300 and $f_{D}≃-1300$ Hz;
- **Approaching:** A concrete truck, at range bin ~330 and $f_{D}≃1100$ Hz, followed by a car that is not appreciated in the RD map.

Table 3 summarizes the quantitative results, in terms of probability of detection $P_{D}$ and maximum detected range bin, for acquisition in scenario 2. Target T1 has a high probability of detection (95.71%) up to maximum range bin 305, when acquisition finished, which translates into a distance of 510 m relative to the PR. The maximum detected range corresponds to target T2 which is detected at a maximum distance of 615 m from the PR. The $P_{D}$ of targets T2–T4 is lower, probably because they are masked in several points, due to the geometry of the area of interest, by target T1, which is nearer at the start of the acquisition.

**Figure 18:**
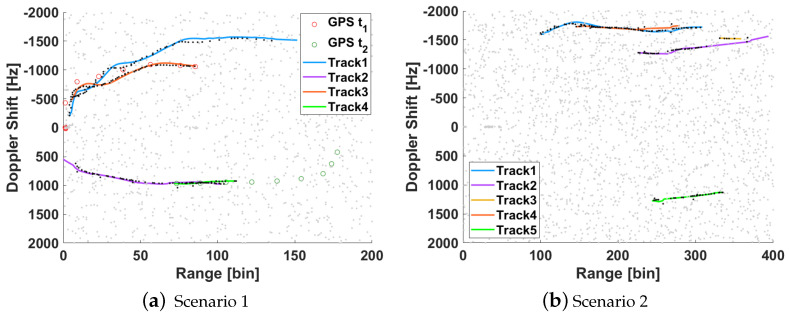
Maps of cumulative detections at the output of CA-CFAR, tracker tracks with their associated detections and GPS ground truth (only for scenario 1).

Accumulated detection matrices generated from the 75 CPIs for both scenario trials are presented in Figure 18 with the estimated tracks and the associated detections in black, the false alarms in grey. For scenario 1, the GPS ground truth for both targets is also represented. In both cases, the number of generated target tracks equals the number of vehicles in the area of interest.

## 7. Conclusions

This work presents the design of an antenna system to improve ground target detection and tracking performances in a DVB-S-based PR. The main challenge is achieving a wide azimuth beamwidth with high gain, which will provide wider coverage than commercial parabolic dish antennas. Reflectarray antennas can be designed for generating contoured beams, but optimization methods must be used for that task. An innovative design method is proposed based on optimizing the position or steering of some partially overlapped beams, instead of optimizing all the phase distribution, for generating a sectorial beam. The different cell element distribution over the reflectarray is optimized by selecting the best phase for the central one. The design procedure is checked by designing a reflectarray antenna suitable for locating it in the structure of a commercial dish antenna, and with challenging requirements for the surveillance channel of a PR. Geometric design variables as well as feed and cell element selection are studied to obtain the best results. The designed reflectarray antenna was simulated in ANSYS HFSS and checked that its radiation pattern fulfilled the requirements of achieving a sectorial beam of $9.4^{\circ }$ in azimuth plane and $5.4^{\circ }$ in elevation, with a maximum gain of 19.13 dBi. A prototype was manufactured and characterized through measurements in an anechoic chamber, proving the simulation results.

The reflectarray was validated for its use as surveillance antenna in PR by carrying out trials in two real terrestrial scenarios with the IDEPAR demonstrator. The real acquisition results fit in really well with previous coverage studies, being able to detect cars at a maximum distance from the PR of almost 600 m in the second deployment scenario. These results show a clear improvement in detection and tracking performances with respect to previously published results.

## Figures and Tables

**Table 1:** Range of phases covered by each element and its associated losses.

Element Type	Phases	Losses
Circle plus triangle	(−180;−125.5]∪[152;180)	$|S_{11}|>-0.6$ dB
Ring $w_{ring}=1.8$ mm	(−125.5;−20]	$|S_{11}|>-3.0$ dB
Ring $w_{ring}=2.8$ mm	(−20;152]	$|S_{11}|>-2.1$ dB

Mean values (*n* = 3 repetitions) preceded by one common letter (a, b) were not significantly different (*p* < 5%).

**Table 2:** Probability of detection of targets in scenario 1 employing the designed reflectarray and a parabolic dish as surveillance antenna. T1–T4 refers to the tracks represented in Figure 18a.

Direction	Parabolic Dish			Reflectarray		
	Target	$P_{D}$	Max. Range	Target	$P_{D}$	Max. Range
Moving away	Car	59.72%	144	Car (T1)	82.67%	151
				Car (T3)	100%	86
Approaching	Bus	79.75%	166	Car (T2)	68%	102
				Car (T4)	83.3%	112

**Table 3:** Probability of detection of targets in scenario 2 employing the designed reflectarray as surveillance antenna. T1–T5 refers to the tracks represented in Figure 18b.

Direction	Reflectarray		
	Target	$P_{D}$	Max. Range [bin]
Moving away	Car (T1)	95.71%	305
	Car (T2)	52.46%	369
	Car (T3)	66.67%	348
	Car (T4)	75%	267
Approaching	Concrete truck (T5)	65.12%	335

## Data Availability

Not applicable.
